# How to measure intraocular pressure: applanation tonometry

**Published:** 2012

**Authors:** Sue Stevens, Clare Gilbert, Nick Astbury

**Affiliations:** Former Nurse Advisor to the *Community Eye Health Journal*: International Centre for Eye Health, London School of Hygiene and Tropical Medicine, London, UK.; Co-Director: International Centre for Eye Health, and Chief Medical Advisor: Sightsavers, UK.; Consultant ophthalmic surgeon (part-time): Norfolk and Norwich University Hospital NHS Trust.

**Figure:**
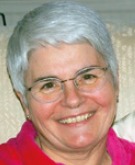
Sue Stevens

**Figure:**
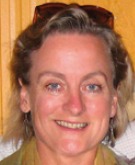
Clare Gilbert

**Figure:**
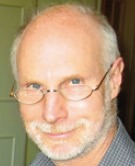
Nick Astbury

Unless there is a contraindication (e.g. trauma or corneal ulcer), all adults attending an eye unit should have their intraocular pressure (IOP) measured. Many people with glaucoma have no symptoms and do not know they have the condition. All children who have had cataract surgery should also have their IOP measured at every follow-up visit, if possible. Finding glaucoma early allows treatment to be given which will preserve sight. Although elevated IOP is not the only sign of glaucoma, measuring it is simple and quick to do. Applanation tonometry, using a Goldmann tonometer at a slit lamp, is the preferred method (the ‘gold standard’).

## Equipment

- Goldmann tonometer
- Applanation prism
- Disinfectant: isopropyl alcohol 70% or sodium hypochlorite 1%
- Local anaesthetic drops
- Fluorescein strips
- Clean cotton wool or gauze swabs.

## Preparation

1. Ensure the prism has been disinfected with isopropyl alcohol 70% or sodium hypochlorite 1%. The prism must be rinsed in sterile water and wiped dry with a clean swab.
  **WARNING**: residue of the disinfectant may cause a caustic burn on the cornea.
2. Check that the gradation marked ‘0’ on the measuring prism is aligned with the white marker point on the tonometer head.
3. Check that the calibrated dial of the tonometer is set around 10 mmHg.
4. Ensure that the patient is sitting comfortably at the slit lamp: at the correct height, with chin on the rest and forehead against the headband.
5. Set the magnification of the slit lamp at × 10.

## Method

**Figure 1:**
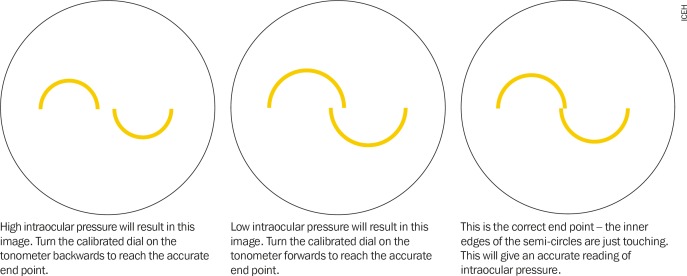
Applanation tonometry semi-circles viewed through the Goldmann prism

**6** Instill the local anaesthetic drops and then the fluorescein. Only a very small amount of fluorescein is needed.
**7** For measuring the IOP in the right eye, make sure the slit beam is shining onto the tonometer head from the patient's right side; for the left eye, the beam should come from the patient's left side.
**8** Move the filters so that the blue filter is used to produce a blue beam.
**9** Make sure the beam of light is as wide as possible, and that the light is as bright as possible. This makes visualising the fluorescein semi-circles easier (with the slit diaphragm fully open).
**10** Ask the patient to look straight ahead, open both eyes wide, and keep perfectly still.
**11** With the thumb, gently hold up the patient's top eyelid, taking care not to put any pressure on the eye
**12** Direct the blue light from the slit lamp onto the prism head.
**13** Make sure that the tonometer head is perpendicular to the eye.
**14** Move the tonometer forward slowly until the prism rests gently on the centre of the patient's cornea.
**15** With the other hand, turn the calibrated dial on the tonometer forward until the two fluorescein semi-circles in the prism head are seen to meet and form a horizontal ‘S’ shape. The correct end point is when the inner edges of the two fluorescein semi-circle images just touch – see Figure 1.
**16** Note the reading on the dial and record it in the notes.
**17** Withdraw the prism from the corneal surface and wipe its tip with a clean, dry swab.
**18** Repeat the procedure for the other eye.
**19** Wipe the prism with a clean, dry swab and replace the prism in the receptacle with just its tip touching the disinfectant.

**Figure:**
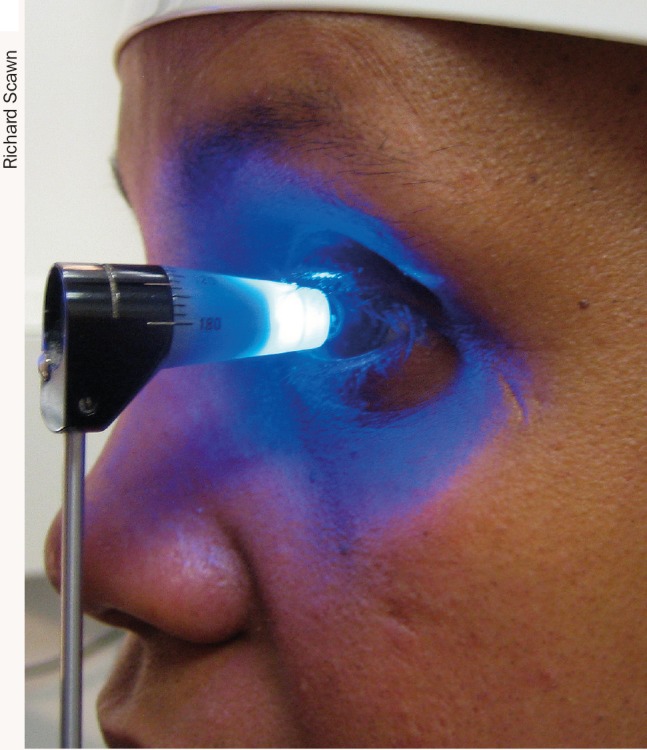
Applanation tonometry. UK

